# Supplementary material for: The efficacy and safety of acetazolamide in chronic mountain sickness: A systematic review and meta‐analysis of randomized controlled trials
Source: PLoS One. 2025 Mar 18;20(3):e0319689. doi: 10.1371/journal.pone.0319689 (PMC11918342; doi:10.1371/journal.pone.0319689)
Supplement: S1 Appendix — (DOCX) [file pone.0319689.s001.docx]

**Pubmed**

Search strategy：

Search: ((((((((((((((((((((((Acetazolamide) OR (Acetazolam)) OR (Acetazolamide Sodium, (Sterile))) OR (Acetazolamide, Monosodium Salt)) OR (Ak-Zol)) OR (Ak Zol)) OR (AkZol)) OR (Apo-Acetazolamide)) OR (Apo Acetazolamide)) OR (ApoAcetazolamide)) OR (Diacarb)) OR (Diamox)) OR (Diuramide)) OR (Défiltran)) OR (Edemox)) OR (Glauconox)) OR (Glaupax)) OR (Huma-Zolamide)) OR (Huma Zolamide)) OR (HumaZolamide)) OR (Acetadiazol)) AND ((Monge's disease) OR (chronic mountain sickness))) AND (("1966/01/01"[Date - Completion] : "2024/08/30"[Date - Completion])) Sort by: Most Recent

("acetazolamide"[MeSH Terms] OR "acetazolamide"[All Fields] OR "acetazolamid"[All Fields] OR ("acetazolamide"[MeSH Terms] OR "acetazolamide"[All Fields]) OR ((("acetazolamide"[MeSH Terms] OR "acetazolamide"[All Fields] OR "acetazolamid"[All Fields]) AND ("sodium"[MeSH Terms] OR "sodium"[All Fields] OR "sodiums"[All Fields])) AND ("infertility"[MeSH Terms] OR "infertility"[All Fields] OR "sterile"[All Fields] OR "sterility"[All Fields] OR "sterilant"[All Fields] OR "sterilants"[All Fields] OR "sterilely"[All Fields] OR "steriles"[All Fields] OR "sterilisation"[All Fields] OR "sterilization, reproductive"[MeSH Terms] OR ("sterilization"[All Fields] AND "reproductive"[All Fields]) OR "reproductive sterilization"[All Fields] OR "sterilization"[All Fields] OR "sterilization"[MeSH Terms] OR "sterilisations"[All Fields] OR "sterilised"[All Fields] OR "steriliser"[All Fields] OR "sterilisers"[All Fields] OR "sterilising"[All Fields] OR "sterilities"[All Fields] OR "sterilise"[All Fields] OR "sterilize"[All Fields] OR "sterilizations"[All Fields] OR "sterilized"[All Fields] OR "sterilizer"[All Fields] OR "sterilizers"[All Fields] OR "sterilizes"[All Fields] OR "sterilizing"[All Fields])) OR ("acetazolamide"[MeSH Terms] OR "acetazolamide"[All Fields] OR ("acetazolamide"[All Fields] AND "monosodium"[All Fields] AND "salt"[All Fields])) OR ("acetazolamide"[MeSH Terms] OR "acetazolamide"[All Fields] OR ("ak"[All Fields] AND "zol"[All Fields])) OR ("acetazolamide"[MeSH Terms] OR "acetazolamide"[All Fields] OR ("ak"[All Fields] AND "zol"[All Fields])) OR ("acetazolamide"[MeSH Terms] OR "acetazolamide"[All Fields]) OR ("acetazolamide"[MeSH Terms] OR "acetazolamide"[All Fields] OR ("apo"[All Fields] AND "acetazolamide"[All Fields])) OR ("acetazolamide"[MeSH Terms] OR "acetazolamide"[All Fields] OR ("apo"[All Fields] AND "acetazolamide"[All Fields])) OR ("acetazolamide"[MeSH Terms] OR "acetazolamide"[All Fields]) OR ("acetazolamide"[MeSH Terms] OR "acetazolamide"[All Fields] OR "diacarb"[All Fields]) OR ("acetazolamide"[MeSH Terms] OR "acetazolamide"[All Fields] OR "diamox"[All Fields]) OR ("acetazolamide"[MeSH Terms] OR "acetazolamide"[All Fields] OR "diuramide"[All Fields]) OR "Defiltran"[All Fields] OR ("acetazolamide"[MeSH Terms] OR "acetazolamide"[All Fields] OR "edemox"[All Fields]) OR ("acetazolamide"[MeSH Terms] OR "acetazolamide"[All Fields]) OR ("acetazolamide"[MeSH Terms] OR "acetazolamide"[All Fields] OR "glaupax"[All Fields]) OR ("acetazolamide"[MeSH Terms] OR "acetazolamide"[All Fields] OR ("huma"[All Fields] AND "zolamide"[All Fields])) OR ("acetazolamide"[MeSH Terms] OR "acetazolamide"[All Fields] OR ("huma"[All Fields] AND "zolamide"[All Fields])) OR ("acetazolamide"[MeSH Terms] OR "acetazolamide"[All Fields]) OR ("acetazolamide"[MeSH Terms] OR "acetazolamide"[All Fields])) AND (("Monge's"[All Fields] AND ("disease"[MeSH Terms] OR "disease"[All Fields] OR "diseases"[All Fields] OR "disease s"[All Fields] OR "diseased"[All Fields])) OR (("chronic"[All Fields] OR "chronical"[All Fields] OR "chronically"[All Fields] OR "chronicities"[All Fields] OR "chronicity"[All Fields] OR "chronicization"[All Fields] OR "chronics"[All Fields]) AND ("altitude sickness"[MeSH Terms] OR ("altitude"[All Fields] AND "sickness"[All Fields]) OR "altitude sickness"[All Fields] OR ("mountain"[All Fields] AND "sickness"[All Fields]) OR "mountain sickness"[All Fields]))) AND 1966/01/01:2024/08/30[Date - Completion]

Translations

Acetazolamide: "acetazolamide"[MeSH Terms] OR "acetazolamide"[All Fields] OR "acetazolamid"[All Fields]

Acetazolam: "acetazolamide"[MeSH Terms] OR "acetazolamide"[All Fields] OR "acetazolam"[All Fields]

Acetazolamide: "acetazolamide"[MeSH Terms] OR "acetazolamide"[All Fields] OR "acetazolamid"[All Fields]

Sodium,: "sodium"[MeSH Terms] OR "sodium"[All Fields] OR "sodiums"[All Fields]

Sterile: "infertility"[MeSH Terms] OR "infertility"[All Fields] OR "sterile"[All Fields] OR "sterility"[All Fields] OR "sterilant"[All Fields] OR "sterilants"[All Fields] OR "sterilely"[All Fields] OR "steriles"[All Fields] OR "sterilisation"[All Fields] OR "sterilization, reproductive"[MeSH Terms] OR ("sterilization"[All Fields] AND "reproductive"[All Fields]) OR "reproductive sterilization"[All Fields] OR "sterilization"[All Fields] OR "sterilization"[MeSH Terms] OR "sterilisations"[All Fields] OR "sterilised"[All Fields] OR "steriliser"[All Fields] OR "sterilisers"[All Fields] OR "sterilising"[All Fields] OR "sterilities"[All Fields] OR "sterilise"[All Fields] OR "sterilize"[All Fields] OR "sterilizations"[All Fields] OR "sterilized"[All Fields] OR "sterilizer"[All Fields] OR "sterilizers"[All Fields] OR "sterilizes"[All Fields] OR "sterilizing"[All Fields]

Acetazolamide, Monosodium Salt: "acetazolamide"[MeSH Terms] OR "acetazolamide"[All Fields] OR ("acetazolamide"[All Fields] AND "monosodium"[All Fields] AND "salt"[All Fields]) OR "acetazolamide, monosodium salt"[All Fields]

Ak-Zol: "acetazolamide"[MeSH Terms] OR "acetazolamide"[All Fields] OR ("ak"[All Fields] AND "zol"[All Fields]) OR "ak zol"[All Fields]

Ak Zol: "acetazolamide"[MeSH Terms] OR "acetazolamide"[All Fields] OR ("ak"[All Fields] AND "zol"[All Fields]) OR "ak zol"[All Fields]

AkZol: "acetazolamide"[MeSH Terms] OR "acetazolamide"[All Fields] OR "akzol"[All Fields]

Apo-Acetazolamide: "acetazolamide"[MeSH Terms] OR "acetazolamide"[All Fields] OR ("apo"[All Fields] AND "acetazolamide"[All Fields]) OR "apo acetazolamide"[All Fields]

Apo Acetazolamide: "acetazolamide"[MeSH Terms] OR "acetazolamide"[All Fields] OR ("apo"[All Fields] AND "acetazolamide"[All Fields]) OR "apo acetazolamide"[All Fields]

ApoAcetazolamide: "acetazolamide"[MeSH Terms] OR "acetazolamide"[All Fields] OR "apoacetazolamide"[All Fields]

Diacarb: "acetazolamide"[MeSH Terms] OR "acetazolamide"[All Fields] OR "diacarb"[All Fields]

Diamox: "acetazolamide"[MeSH Terms] OR "acetazolamide"[All Fields] OR "diamox"[All Fields]

Diuramide: "acetazolamide"[MeSH Terms] OR "acetazolamide"[All Fields] OR "diuramide"[All Fields]

Edemox: "acetazolamide"[MeSH Terms] OR "acetazolamide"[All Fields] OR "edemox"[All Fields]

Glauconox: "acetazolamide"[MeSH Terms] OR "acetazolamide"[All Fields] OR "glauconox"[All Fields]

Glaupax: "acetazolamide"[MeSH Terms] OR "acetazolamide"[All Fields] OR "glaupax"[All Fields]

Huma-Zolamide: "acetazolamide"[MeSH Terms] OR "acetazolamide"[All Fields] OR ("huma"[All Fields] AND "zolamide"[All Fields]) OR "huma zolamide"[All Fields]

Huma Zolamide: "acetazolamide"[MeSH Terms] OR "acetazolamide"[All Fields] OR ("huma"[All Fields] AND "zolamide"[All Fields]) OR "huma zolamide"[All Fields]

HumaZolamide: "acetazolamide"[MeSH Terms] OR "acetazolamide"[All Fields] OR "humazolamide"[All Fields]

Acetadiazol: "acetazolamide"[MeSH Terms] OR "acetazolamide"[All Fields] OR "acetadiazol"[All Fields]

disease: "disease"[MeSH Terms] OR "disease"[All Fields] OR "diseases"[All Fields] OR "disease's"[All Fields] OR "diseased"[All Fields]

chronic: "chronic"[All Fields] OR "chronical"[All Fields] OR "chronically"[All Fields] OR "chronicities"[All Fields] OR "chronicity"[All Fields] OR "chronicization"[All Fields] OR "chronics"[All Fields]

mountain sickness: "altitude sickness"[MeSH Terms] OR ("altitude"[All Fields] AND "sickness"[All Fields]) OR "altitude sickness"[All Fields] OR ("mountain"[All Fields] AND "sickness"[All Fields]) OR "mountain sickness"[All Fields]
