# Supplementary material for: The efficacy and safety of acetazolamide in chronic mountain sickness: A systematic review and meta‐analysis of randomized controlled trials
Source: PLoS One. 2025 Mar 18;20(3):e0319689. doi: 10.1371/journal.pone.0319689 (PMC11918342; doi:10.1371/journal.pone.0319689)
Supplement: S1 Table — (DOCX) [file pone.0319689.s005.docx]

**S1 Table.** GRADE quality of evidence assessment for each outcome.

| **Outcome** | **Limitations** | **Inconsistency** | **Indirectness** | **Imprecision** | **Publication bias** | **N of participants** | **Conclusion** | **Quality of evidence** |
| --- | --- | --- | --- | --- | --- | --- | --- | --- |
| **Primary outcome** |  |  |  |  |  |  |  |  |
| CMS clinical score [26,27,29,30] | Small sample size ^a^ | No | No | No | Undetected | 105 | No difference | Low quality (⊕⊕OO) |
| **Secondary outcomes** |  |  |  |  |  |  |  |  |
| CMS total score[26-28] | Small sample size ^a^ | No | No | No | Undetected | 71 | ACZ superior to placebo | Low quality (⊕⊕OO) |
| HCT[26-30] | Small sample size ^a^ | No | No | No | Undetected | 137 | ACZ superior to placebo | Low quality (⊕⊕OO) |
| PaO2[26-30] | Small sample size ^a^ | No | No | No | Undetected | 137 | ACZ superior to placebo | Low quality (⊕⊕OO) |
| PaCO2[26-30] | Small sample size ^a^ | No | No | No | Undetected | 137 | ACZ superior to placebo | Low quality (⊕⊕OO) |
| pH[26-30] | Small sample size ^a^ | Serious ^b^ | No | No | Undetected | 137 | ACZ superior to placebo | Very low quality (⊕OOO) |
| HCO3[27-30] | Small sample size ^a^ | Serious ^b^ | No | No | Undetected | 110 | ACZ superior to placebo | Very low quality (⊕OOO) |
| **Adverse Events** |  |  |  |  |  |  |  |  |
| Increased Diuresis[29,30] | Small sample size ^a^ | No | No | No | Undetected | 66 | No difference | Low quality (⊕⊕OO) |
| Paresthesia[28-30] | Small sample size ^a^ | No | No | No | Undetected | 98 | ACZ inferior to placebo | Low quality (⊕⊕OO) |
| Headache[28,29] | Small sample size ^a^ | No | No | No | Undetected | 79 | No difference | Low quality (⊕⊕OO) |

N, number; ACZ,acetazolamide.

a Five or less trials reported these outcomes. Final decision to rate down quality of evidence by two levels for serious limitation.

b I2 was above 50% with wide variance of point estimates across studies. Final decision to rate down quality of evidence by one level for moderate or serious inconsistency.
