# Supplementary material for: The efficacy and safety of acetazolamide in chronic mountain sickness: A systematic review and meta‐analysis of randomized controlled trials
Source: PLoS One. 2025 Mar 18;20(3):e0319689. doi: 10.1371/journal.pone.0319689 (PMC11918342; doi:10.1371/journal.pone.0319689)
Supplement: Attachment — Submitted filename: Response to Reviewers.docx [file pone.0319689.s007.docx]

PONE-D-24-42748

The efficacy and safety of acetazolamide in chronic mountain sickness：a systematic review and meta‐analysis of randomized controlled trials

PLOS ONE

Dear Dr. Feng,

Thank you for submitting your manuscript to PLOS ONE. After careful consideration, we feel that it has merit but does not fully meet PLOS ONE’s publication criteria as it currently stands. Therefore, we invite you to submit a revised version of the manuscript that addresses the points raised during the review process.

Please submit your revised manuscript by Jan 25 2025 11:59PM. If you will need more time than this to complete your revisions, please reply to this message or contact the journal office at plosone@plos.org. When you're ready to submit your revision, log on to https://www.editorialmanager.com/pone/ and select the 'Submissions Needing Revision' folder to locate your manuscript file.

Please include the following items when submitting your revised manuscript:

A rebuttal letter that responds to each point raised by the academic editor and reviewer(s). You should upload this letter as a separate file labeled 'Response to Reviewers'.

A marked-up copy of your manuscript that highlights changes made to the original version. You should upload this as a separate file labeled 'Revised Manuscript with Track Changes'.

An unmarked version of your revised paper without tracked changes. You should upload this as a separate file labeled 'Manuscript'.

If you would like to make changes to your financial disclosure, please include your updated statement in your cover letter. Guidelines for resubmitting your figure files are available below the reviewer comments at the end of this letter.

If applicable, we recommend that you deposit your laboratory protocols in protocols.io to enhance the reproducibility of your results. Protocols.io assigns your protocol its own identifier (DOI) so that it can be cited independently in the future. For instructions see: https://journals.plos.org/plosone/s/submission-guidelines#loc-laboratory-protocols. Additionally, PLOS ONE offers an option for publishing peer-reviewed Lab Protocol articles, which describe protocols hosted on protocols.io. Read more information on sharing protocols at https://plos.org/protocols?utm_medium=editorial-email&utm_source=authorletters&utm_campaign=protocols.

We look forward to receiving your revised manuscript.

Kind regards,

Tungki Pratama Umar, M.D.

Academic Editor

PLOS ONE

**Response：**

**Dear Editors and Reviewers:**

Thank you for your letter and for the reviewers’ comments concerning our manuscript entitled “**The efficacy and safety of acetazolamide in chronic mountain sickness：a systematic review and meta‐analysis of randomized controlled trials**”.Those comments are all valuable and very helpful for revising and improving our paper,as well as the important guiding significance to our researches.We have studied comments carefully and have made correction which we hope meet with approval.The main corrections in paper and the responds to the editorial corrections and reviewer’s comments are as flowing:

Journal Requirements:

When submitting your revision, we need you to address these additional requirements.

1. Please ensure that your manuscript meets PLOS ONE's style requirements, including those for file naming. The PLOS ONE style templates can be found at

https://journals.plos.org/plosone/s/file?id=wjVg/PLOSOne_formatting_sample_main_body.pdf and

https://journals.plos.org/plosone/s/file?id=ba62/PLOSOne_formatting_sample_title_authors_affiliations.pdf

**Response：Thank you for your suggestion.I have carefully read the website you provided and made corresponding modifications to the manuscript according to its requirements, ensuring that it meets the style requirements of your journal.**

2. As required by our policy on Data Availability, please ensure your manuscript or supplementary information includes the following:

A numbered table of all studies identified in the literature search, including those that were excluded from the analyses.

For every excluded study, the table should list the reason(s) for exclusion.

If any of the included studies are unpublished, include a link (URL) to the primary source or detailed information about how the content can be accessed.

A table of all data extracted from the primary research sources for the systematic review and/or meta-analysis. The table must include the following information for each study:

Name of data extractors and date of data extraction

Confirmation that the study was eligible to be included in the review.

All data extracted from each study for the reported systematic review and/or meta-analysis that would be needed to replicate your analyses.

If data or supporting information were obtained from another source (e.g. correspondence with the author of the original research article), please provide the source of data and dates on which the data/information were obtained by your research group.

If applicable for your analysis, a table showing the completed risk of bias and quality/certainty assessments for each study or outcome. Please ensure this is provided for each domain or parameter assessed. For example, if you used the Cochrane risk-of-bias tool for randomized trials, provide answers to each of the signalling questions for each study. If you used GRADE to assess certainty of evidence, provide judgements about each of the quality of evidence factor. This should be provided for each outcome.

An explanation of how missing data were handled.

This information can be included in the main text, supplementary information, or relevant data repository. Please note that providing these underlying data is a requirement for publication in this journal, and if these data are not provided your manuscript might be rejected.

**Response：Thank you for your suggestion.As per your request, I have provided the corresponding supplementary materials and data, please refer to the materials I provided this time for details.**

3. Thank you for stating the following financial disclosure:

"The present study was supported by the Project for the First Hospital Fund of Lanzhou University (grant no. ldyyyn2023-56)."

Please state what role the funders took in the study. If the funders had no role, please state: ""The funders had no role in study design, data collection and analysis, decision to publish, or preparation of the manuscript.""

If this statement is not correct you must amend it as needed.

Please include this amended Role of Funder statement in your cover letter; we will change the online submission form on your behalf.

**Response：Thank you for your suggestion.The funders play a leading role in research design, data collection and analysis, publication decisions, and manuscript preparation.**

4. Thank you for stating the following in the Acknowledgments Section of your manuscript:

"The authors gratefully acknowledge all the authors who shared the results of the included studies, the support of the Research Center for Clinical Medicine,The First Hospital of Lanzhou University.The present study was supported by the Project for the First Hospital Fund of Lanzhou University (grant no. ldyyyn2023-56)."

We note that you have provided funding information that is not currently declared in your Funding Statement. However, funding information should not appear in the Acknowledgments section or other areas of your manuscript. We will only publish funding information present in the Funding Statement section of the online submission form.

Please remove any funding-related text from the manuscript and let us know how you would like to update your Funding Statement. Currently, your Funding Statement reads as follows:

"The present study was supported by the Project for the First Hospital Fund of Lanzhou University (grant no. ldyyyn2023-56)."

Please include your amended statements within your cover letter; we will change the online submission form on your behalf.

**Response：Thank you for your suggestion.As per your request, I have removed the fund information from the manuscript and will provide a detailed explanation in the cover letter.**

5. We note that your Data Availability Statement is currently as follows: All relevant data are within the manuscript and its Supporting Information files.

Please confirm at this time whether or not your submission contains all raw data required to replicate the results of your study. Authors must share the “minimal data set” for their submission. PLOS defines the minimal data set to consist of the data required to replicate all study findings reported in the article, as well as related metadata and methods (https://journals.plos.org/plosone/s/data-availability#loc-minimal-data-set-definition).

For example, authors should submit the following data:

- The values behind the means, standard deviations and other measures reported;

- The values used to build graphs;

- The points extracted from images for analysis

Authors do not need to submit their entire data set if only a portion of the data was used in the reported study.

If your submission does not contain these data, please either upload them as Supporting Information files or deposit them to a stable, public repository and provide us with the relevant URLs, DOIs, or accession numbers. For a list of recommended repositories, please see https://journals.plos.org/plosone/s/recommended-repositories.

If there are ethical or legal restrictions on sharing a de-identified data set, please explain them in detail (e.g., data contain potentially sensitive information, data are owned by a third-party organization, etc.) and who has imposed them (e.g., an ethics committee). Please also provide contact information for a data access committee, ethics committee, or other institutional body to which data requests may be sent. If data are owned by a third party, please indicate how others may request data access.

**Response：Thank you for your valuable suggestion.As per your request, I have provided detailed raw materials and data in this submission.**

6. Please include a separate caption for each figure in your manuscript.

**Response：Thank you for your review.We have made modifications according to your request.**

7. Please include captions for your Supporting Information files at the end of your manuscript, and update any in-text citations to match accordingly. Please see our Supporting Information guidelines for more information: http://journals.plos.org/plosone/s/supporting-information.

**Response：Thank you very much for your proposal. I have carefully read the relevant requirements and made corresponding modifications.**

Academic Editor Comments:

Title

1. Please see my comment based on RCT-only study (result and miscellaneous).

Suggested title:

The efficacy and safety of acetazolamide in chronic mountain sickness：a systematic review and meta‐analysis of randomized placebo-controlled clinical trials

**Response：Thank you very much for your proposal.But according to our research purpose, we did not make any modifications to the title.**

Abstract

1. It should include a background information, consider seeing other published systematic review articles at Plos One

2. Result section for insignificant result can be delivered concisely, not to specific as in the current version

3. It is better to add that more clinical trials with more participants are anticipated to further validated the findings (currently it is too

**Response：Thank you very much for your review and valuable suggestions. I have optimized the abstract section based on all your suggestions.**

Introduction

1. erythrocytosis (EE) - should be excessive erythrocytosis (EE)

2. ....and an increase in hematocrit (Hct > 63%) is not universally adapted for everyone, it should be classified also as lower in women (approximately three times of Hb value) (https://www.frontiersin.org/journals/physiology/articles/10.3389/fphys.2020.00773/full)

3. Consider breaking and editing first paragraph of the introduction to two paragraphs, first one to describe CMS and second to inform about CMS cases

4. Third paragraph should describe available treatment options and describing acetazolamide role, also describing in more detail what is "there remains a lack of solid evidence-based support for its use" although it is already approved by the FDA

**Response：**

**1.Thank you for your suggestion. I have made the necessary modifications.**

**2.Thank you very much for your suggestions and reference materials provided. I have carefully read the literature and made corresponding modifications to the manuscript.**

**3.Thank you for your suggestion. I have made the necessary modifications.**

Methods

1. Add PRISMA long form and cite it

2. Add whether there is any age limitation or adult only inclusion in this study, also define what is "extended" (minimum period to be considered as a CMS)

3. Add any other types of human study, such as observational study (case-control, cohort, cross-sectional), case report, case series, etc

4. Cite which Endnote version that was used in this study

5. Cite Cochrane RoB tool utilized in this study, also Revman version

6. Add the reference stating I^2 50% as the threshold of heterogenous or homogenous data

**Response：**

**1.Thank you for your suggestion. PRISMA long form has been added.**

**2.Thank you for your suggestion.We limit it to adults who have lived in high-altitude areas for at least 1 year.**

**3.Thank you very much for your suggestion, but my initial inclusion criteria were to conduct Meta-analysis based on RCTs, so no other types of studies were added.**

**4.Added corresponding version information.**

**5.Added corresponding version information and referenced ROB tools based on this version.**

**6.We interpreted I2 using the guidance available from the Cochrane handbook.**

Result

1. Since the authors stated that there is no blinding in reference 27, it cannot be considered as an RCT (just an experimental study, likely a quasi-experimental study), reconsider its inclusion

2. Divide its subsection based on similarity, such as laboratory parameters (pH, PaO2), etc

3. I agreed with reviewer 2 comments (why HCT is the primary outcome, not clinical score), please elaborate more or revise accordingly

4. Please do the GRADE analysis since the authors have strongly claimed that "we conclude that ACZ 250 mg is a safe, reliable, and low-cost treatment option for chronic mountain sickness. By reducing HCT, PaCO2, pH, and HCO3, and increasing PaO2, it improves respiratory and circulatory parameters in CMS patients and effectively treats CMS."

5. In accordance with a relatively high risk of bias in reference 27, it is recommended to remove it from the analysis (also because it is not RCT)

**Response：**

**1.Thank you for your suggestion. Although this study did not use blinding, it indicates that it is a low-quality randomized controlled trial. Therefore, after careful consideration, we believe that it meets our inclusion criteria.**

**2.Thank you very much for your review,We have already added laboratory parameters group as per your request.**

**3.Thank you very much for your review and valuable suggestions. I have revised the CMS clinical score to the primary outcome measure based on all your suggestions。**

**4.Thank you for your suggestion. We have provided a GRADE analysis and evaluation form in the supplementary materials.**

**5.Thank you very much for your review.Despite the high risk of bias in reference 27, it is still an RCT study and, after careful consideration, we believe it meets our inclusion criteria.**

Discussion

1. Revise this: This study includes all "randomized controlled literature"

2. Please discuss more about the relevance of laboratory parameters and clinical status amelioration of CMS patients

3. Delete last sentence, it is redundant with the conclusion section (can be considered to be merged with it)

**Response：**

**1.Thank you very much for your review and valuable suggestions. We have made corresponding modifications based on this.**

**2.Thank you for your suggestion. I have made the necessary modifications.**

**3.Thank you for your suggestion. I deleted the last sentence as per your request.**

Conclusion

1. Same as abstract (3), basically to add further clinical trials or any other issues that should be encountered to strengthen the evidence

**Response：**

**1.Thank you for your suggestion. I have made the necessary modifications.**

Table

1. Revise extraction table, it is distorted in the current version.

**Response：**

**1.Thank you for your suggestion. I have made the necessary modifications.**

Miscellaneous

1. If the authors want to keep their statement in the title: The efficacy and safety of acetazolamide in chronic mountain sickness：a systematic review and meta‐analysis of randomized controlled trials, the authors should remove a study with reference 27 (it is not an RCT)

**Response：**

**1.Thank you very much for your review.Despite the high bias in reference 27, it is still an RCT study and after careful examination by our team, we believe it meets our inclusion criteria.**

Citation

1. The authors should consider to make the references using referencing software, it is observed that there are several issues such as this: [26][27][28][29][30], which should be made as [26-30] AND writing of some journals such as J Appl pHysiol or Exp pHysiol, indicating inconsistent capitalization

**Response：**

**1.Thank you for your suggestion. I have made the necessary modifications.**

[Note: HTML markup is below. Please do not edit.]

Reviewers' comments:

Reviewer's Responses to Questions

Comments to the Author

1. Is the manuscript technically sound, and do the data support the conclusions?

The manuscript must describe a technically sound piece of scientific research with data that supports the conclusions. Experiments must have been conducted rigorously, with appropriate controls, replication, and sample sizes. The conclusions must be drawn appropriately based on the data presented.

Reviewer #1: Yes

Reviewer #2: Partly

Reviewer #3: Yes

**Response：Thank you very much for your review and approval.**

2. Has the statistical analysis been performed appropriately and rigorously?

Reviewer #1: Yes

Reviewer #2: Yes

Reviewer #3: Yes

**Response：Thank you very much for your review and approval.**

3. Have the authors made all data underlying the findings in their manuscript fully available?

The PLOS Data policy requires authors to make all data underlying the findings described in their manuscript fully available without restriction, with rare exception (please refer to the Data Availability Statement in the manuscript PDF file). The data should be provided as part of the manuscript or its supporting information, or deposited to a public repository. For example, in addition to summary statistics, the data points behind means, medians and variance measures should be available. If there are restrictions on publicly sharing data—e.g. participant privacy or use of data from a third party—those must be specified.

Reviewer #1: Yes

Reviewer #2: Yes

Reviewer #3: Yes

**Response：Thank you very much for your review and approval.**

4. Is the manuscript presented in an intelligible fashion and written in standard English?

PLOS ONE does not copyedit accepted manuscripts, so the language in submitted articles must be clear, correct, and unambiguous. Any typographical or grammatical errors should be corrected at revision, so please note any specific errors here.

Reviewer #1: Yes

Reviewer #2: Yes

Reviewer #3: Yes

**Response：Thank you very much for your review and approval.**

5. Review Comments to the Author

Please use the space provided to explain your answers to the questions above. You may also include additional comments for the author, including concerns about dual publication, research ethics, or publication ethics. (Please upload your review as an attachment if it exceeds 20,000 characters)

Reviewer #1: The efficacy and safety of acetazolamide in chronic mountain sickness：a systematic review and meta‐analysis of randomized controlled trials

A well performed and meticulous Meta-analysis of the effect of short term ACZ treatment of CMS.

Unfortunately, CMS is currently not clearly understood as it is assumed to be an isolated single disease present in some individuals at high altitude. Quite the contrary, CMS is in reality multiple diseases pulmonary, cardiac, renal, carotid body, hypoventilation and other alterations in a chronic hypoxia environment. Hence to treat all these multiple diseases as a sole individual pathology at high altitude gives rise to inadequate treatments. A poor diagnosis in medicine gives rise to ineffective and even deleterious effects of pharmaceutical strategies.

In order to adequately interpret CMS it is important to read “Redefining chronic mountain sickness: insights from high-altitude research and clinical experience”.

https://doi.org/10.1515/mr-2024-0036

In this Meta-analysis article based on the consensus statement CMS is “defined as hemoglobin concentration [Hb] ≥ 21 g/dL in men and ≥ 19 g/dL in women) and an increase in hematocrit (Hct > 63%), resulting in significantly increased blood viscosity, microcirculation disturbances, and even widespread organ damage and reduced blood flow velocity”

This is a misconception due to the poor understanding of CMS from the medical point of view as shown in the article above.

Approximately 81.6 million people worldwide live in high-altitude regions (>2500 meters) is not precise and should be reviewed as several papers point out to even over 100 million people.

Table 1 is disorganized (by software) and difficult to understand.

It is perfectly understandable that ACZ could not improve the clinical symptoms of CMS patients Fig. 4.

The results confirm ACZ effects but it questionable if it is a solution to CMS. Making pH acidic is not a good physiological response as cells require an optimal pH to function.

In the adverse events it becomes evident that the Richalet study was biased as compared to the other 4 studies, as no specific data is provided. Furthermore, upon literature review it is mentioned that in that study by Richalet there was actually a death episode, that was questionably attributed to other circumstances but this needs to be clearly stated.

Parestesia is indeed an important side effect of those using ACZ. It is very uncomfortable and often a sign of complain by those using it to prevent AMS.

The important analyses and evidence based observations regarding an increase of PV with the use of ACZ, gives rise to the question of confounding artificial benefits in the treatments.

with reference to “a new clinical CMS scoring system should be developed for interventional studies” it is evident that CMS scoring system is not efficient as it is not based in the true understanding of CMS.

Furthermore, the current treatment duration was relatively short, and it is highly questionable for long term use. Once the treatment is stopped after short use, the patients with CMS will return to their normal condition of increased Ht, lower PaO2, higher PaCO2 as this is a normal adaptive physiological response to disease in the chronic hypoxia environment.

In conclusion this excellent meta-analysis is actually a report of 5 different studies based on a wrong assumption of the short term treatment with ACZ of CMS interpreted as a sole entity with expected pharmacological results of the use of this inhibitor of carbonic anhydrase. In other words, it is excellent to show the effect of ACZ on the organism but questionable in its use as a permanent treatment of what is wrongly defined as CMS.

As the authors correctly mention in the limitations section, “more high-quality, large-sample, multi-center randomized studies are needed in the future to further validate the efficacy and safety of different doses of ACZ and long-term treatment for CMS”. It is highly probable that such a long term large population study will have unfavorable outcomes as ACZ is creating a pharmacological confounding response that satisfies the desperate attempt to reduce the Ht without a true understanding that the increase of Ht is actually a life saving physiological response to organ insufficiencies of multiple disease at high altitude. It may also have negative effects on longevity within those diseases at high altitudes.

**Response:Thank you very much for your review.We have made comprehensive corrections based on the issues you mentioned above.**

**Answer to viewpoint questions,I have carefully and attentively read the literature you provided, which has taught me more about CMS. I also strongly agree with your point of view that the diagnosis, treatment, and scoring systems of CMS have certain limitations.I will pay more attention to the in-depth understanding of CMS in future research, although the diagnostic criteria for CMS have not changed yet.**

Reviewer #2: The purpose of this systematic review was to evaluate the effectiveness and safety of acetazolamide(ACZ) in the treatment of chronic mountain sickness (CMS). The results of five randomized controlled trials were included, comprising a total of 137 subjects, with in the acetazolamide group and in the control group. The results show that ACZ 250 mg is a safe, reliable, and low-cost treatment option for chronic mountain sickness. It reduces hematocrit, arterial blood carbon dioxide pressure, pH, and bicarbonate concentration as well as increases arterial blood oxygen pressure as well as clinical scores of chronic mountain sickness.

The topic and the results are of interest, however, there are also some important concerns to be addressed.

1. Abstract/Methods, pH is the measure of H+ ion concentration, not the “potential of hydrogen”. The text should be revised accordingly.

2. Introduction, “oxygen receptors in the kidney” – I suppose authors mean hypoxia-induced factor (HIF). Strictly speaking, HIF should not be considered as the oxygen receptor because oxygen regulates HIF by modifying its hydroxylation through prolyl hydroxylase. Because O2 does not bind to HIF, the latter should not be named oxygen receptor.

3. Page 12, lines 1-4, the text suggests that ACZ has several mechanisms of action whereas all the effects listed here result from inhibiting carbonic anhydrase. The role of the enzyme in acid-base balance should be briefly decribed as well as the mechanisms through which its inhibition results in all effects listed in the sentence.

4. What are the specific aims of this meta-analysis? Meta-analyses are usually performed if there are discrepancies between the results of various experimental studies. No such discrepancies are mentioned in the Introduction and the rationale of performing the meta-analysis is questionable.

5. Exclusion criteria #1 – please specify which “other acute or chronic illnesses” were excluded and what approach was used for this purpose.

6. Why hematocrit rather than clinical score of CMS was the primary endpoint of interest?

7. Is there any relationship between duration of ACZ therapy and the outcomes?

**Response:Thank you very much for your review and agreement.**

**1.Modified according to your requirements.**

**2.Modified according to your requirements.**

**3.The mechanism of action of ACZ is introduced in the introduction section.**

**4.As mentioned in my article,the purpose of this systematic review is to evaluate the effectiveness and safety of acetazolamide in the treatment of chronic mountain sickness and this study provides the first evidence-based evidence for ACZ treatment of CMS.**

**5.All other acute and chronic diseases mentioned by us are excluded (including any organ dysfunction, acute or chronic injury, infection, etc.), and these information sources are based on the original literature.**

**6.Based on your feedback, I have identified CMS clinical score as the primary outcome.**

**7.In this study, due to limited raw data, we did not investigate the relationship between treatment duration and outcomes. If possible, we will investigate this in future studies.**

Reviewer #3: I read with interest the manuscript entitled “The efficacy and safety of acetazolamide in chronic mountain sickness: a systematic review and meta‐analysis of randomized controlled trials”. The manuscript is well written and address an issue of interest with few quality data available.

**Response:Thank you very much for your review and agreement.**

Major comments

Inclusion criteria might include acetazolamide dose of 250mg/day

**Response:Thank you very much for your suggestion, but I don't think it is necessary or the best choice to include ACZ dose of 250mg/day.**

Length of treatment was not analyzed in depth. Inclusion criteria state that patients who have lived at high altitudes (≥2500 m) for an “extended period” were included. How long is an “extended period”

**Response:Based on the included literature, we set it as no less than 1 year.**

Length of treatment may also impact on some of the negative outcomes; therefore, it would be nice to have detailed median lengths of treatment by study.

**Response:I completely agree with your viewpoint. Due to limited data, we will consider this matter in future research。**

The score (CMS) is composed of many variables, it would be possible that Acetazolamide may have beneficial effects only in some.

**Response:I completely agree with your viewpoint.**

It would also be of importance to know if results can be sustained enough in time to revert cardiovascular adaptation. How long were the patients followed before assessing outcomes?

**Response:In the original literature, the treatment duration for most studies was 3 weeks, but there were also 6 weeks and 12 weeks. Due to limited data, no data analysis was conducted。**

Minor comments

Document is not paginated

There are some mistakes in table 1 with headings in page 14 and body in the following page. The legend of table 1, is also missed in page 14.

**Response:This error has been corrected。**

6. PLOS authors have the option to publish the peer review history of their article (what does this mean?). If published, this will include your full peer review and any attached files.

If you choose “no”, your identity will remain anonymous but your review may still be made public.

Do you want your identity to be public for this peer review? For information about this choice, including consent withdrawal, please see our Privacy Policy.

Reviewer #1: Yes: Gustavo Zubieta-Calleja

Reviewer #2: No

Reviewer #3: No

[NOTE: If reviewer comments were submitted as an attachment file, they will be attached to this email and accessible via the submission site. Please log into your account, locate the manuscript record, and check for the action link "View Attachments". If this link does not appear, there are no attachment files.]

While revising your submission, please upload your figure files to the Preflight Analysis and Conversion Engine (PACE) digital diagnostic tool, https://pacev2.apexcovantage.com/. PACE helps ensure that figures meet PLOS requirements. To use PACE, you must first register as a user. Registration is free. Then, login and navigate to the UPLOAD tab, where you will find detailed instructions on how to use the tool. If you encounter any issues or have any questions when using PACE, please email PLOS at figures@plos.org. Please note that Supporting Information files do not need this step.
